# Cortical superficial siderosis, hematoma volume, and outcomes after intracerebral hemorrhage: a mediation analysis

**DOI:** 10.3389/fneur.2023.1122744

**Published:** 2023-05-05

**Authors:** Yu-jia Jin, Jia-wen Li, Jian Wu, Yu-hui Huang, Kai-cheng Yang, Hong-na An, Chang-zheng Yuan, Feng Gao, Lu-sha Tong

**Affiliations:** ^1^Department of Neurology, The 2nd Affiliated Hospital of Zhejiang University, School of Medicine, Hangzhou, China; ^2^School of Public Health, Zhejiang University School of Medicine, Hangzhou, China; ^3^Department of Neurology, The 2nd People's Hospital of Quzhou, Quzhou, China

**Keywords:** cSS, ICH volume and outcome, mediation analysis, prospective studies, cerebral hemorrhage, hematoma

## Abstract

**Background:**

Previous studies have shown that cortical superficial siderosis (cSS) can increase hematoma volume and predict poor outcomes following primary intracerebral hemorrhage (ICH).

**Objective:**

We aimed to determine whether a large hematoma volume was the essential factor contributing to worse outcomes of cSS.

**Methods:**

Patients with spontaneous ICH underwent a CT scan within 48 h after ictus. Evaluation of cSS was performed using magnetic resonance imaging (MRI) within 7 days. The 90-day outcome was assessed using the modified Rankin Scale (mRS). In addition, we investigated the correlation between cSS, hematoma volume, and 90-day outcomes using multivariate regression and mediation analyses.

**Results:**

Among the 673 patients with ICH [mean (SD) age, 61 (13) years; 237 female subjects (35.2%); median (IQR) hematoma volume, 9.0 (3.0–17.6) ml], 131 (19.5%) had cSS. There was an association between cSS and larger hematoma volume (β = 4.449, 95% CI 1.890–7.009, *p* < 0.001) independent of hematoma location and was also related to worse 90-day mRS (β = 0.333, 95% CI 0.008–0.659, *p* = 0.045) in multivariable regression. In addition, mediation analyses revealed that hematoma volume was an essential factor mediating the effect of cSS on unfavorable 90-day outcomes (proportion mediated:66.04%, *p* = 0.01).

**Conclusion:**

Large hematoma volume was the major charge of directing cSS to worse outcomes in patients with mild to moderate ICH, and cSS was related to a larger hematoma in both lobar and non-lobar areas.

**Clinical trial registration:**

https://clinicaltrials.gov/ct2/show/NCT04803292, identifier: NCT04803292.

## 1. Introduction

Cortical superficial siderosis (cSS) has been defined as the previous extravasation of blood on the superficial layers of the cortex or subarachnoid space, which can be detected as a hypointense curvilinear signal on the susceptibility-weighted image (SWI) on magnetic resonance imaging (MRI) (1, 2). Observations from large cohorts revealed that patients with cSS were more likely to have large intracerebral hemorrhage (ICH) volume and hematoma expansion, especially in the lobar area. cSS was also reported to correlate with poor outcomes after ICH (1, 3–8). However, whether cSS leads to worse outcomes solely by itself or through a specific pathway (especially hematoma volume) and whether cSS-related enlarged hematoma is restricted to the lobar area because of the origin of cSS remain unknown. A better understanding of this relationship could provide evidence of the latent mechanisms by which cSS induces hemorrhage. In this context, we extrapolated several factors that might be the essential factors mediating the association between cSS and worse outcomes, and we tested the relationship between cSS and hematoma volume separately in lobar and non-lobar areas.

## 2. Materials and methods

### 2.1. Study design

This study collected data from consecutively enrolled patients with ICH from a single center (Department of Neurology, the Second Affiliated Hospital of Zhejiang University, Hangzhou) between November 2016 and February 2021 (ClinicalTrials.gov Identifier: NCT 04803292). The cohort included patients who had an initial diagnosis of primary ICH in the emergency room (according to their past medical history and emergency examinations) and did not undergo emergency surgery. This study was approved by the Human Ethics Committee of the Second Affiliated Hospital of Zhejiang University and followed the tenets specified in the 1975 Declaration of Helsinki. Written informed consent was obtained from all participants.

### 2.2. Patient inclusion and exclusion

Patients ≥ 18 years of age admitted to the hospital within 48 h of primary ICH were included. Suspected secondary ICH caused by trauma, aneurysm or vascular malformation, systemic disease-related coagulopathy, hemorrhagic venous infarct, or hemorrhagic transformation of ischemic stroke was omitted. The diagnoses were determined by experienced neurologists based on medical history and neuroimaging findings. Patients with isolated intraventricular hemorrhage (IVH) and those who underwent neurosurgical procedures were also excluded.

**Figure 1:**
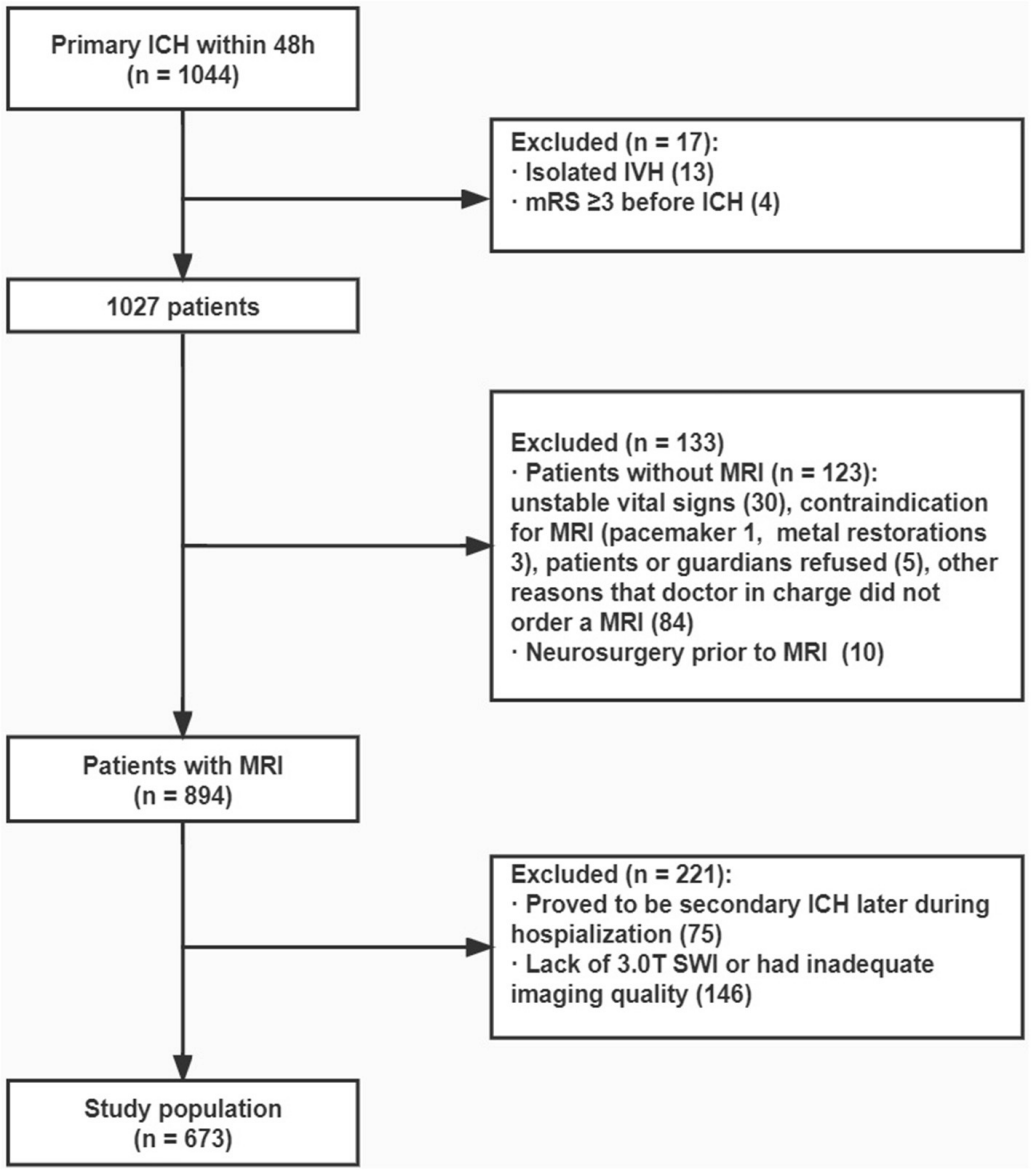
Flowchart. From the original cohort of 1,044 patients with primary ICH that occurred within 48 h, 673 patients were finally included in analysis.

We included patients who underwent a 3.0 T MRI examination with SWI sequences after ICH ictus for this study focusing on cSS. In addition, patients with a modified Rankin scale (mRS) score of ≥3 before ICH (according to medical records or inquiries to relatives) were excluded (Figure 1).

### 2.3. Data collection

We prospectively collected the following information: (i) demographic characteristics, including age and sex; (ii) presence of vascular risk factors and medical history (smoking, alcohol intake, hypertension, diabetes mellitus, coronary heart disease, ischemic stroke or TIA, ICH) according to the description from the patients or their relatives, or medical records using previously published definitions; (9) (iii) atrial fibrillation based on the electrocardiogram obtained during hospitalization or previous medical records; (iv) medication at the baseline, including antiplatelet drugs, anticoagulant drugs, and statins; and (v) Glasgow Coma Score (GCS) on admission.

### 2.4. Assessment of hematoma volume

Patients underwent a non-contrast CT scan at admission within 48 h after ictus on multidetector row scanners (Optima CT540, General Electric Healthcare, Connecticut, USA; or SOMATOM Sensation 16, Siemens, Germany) with the following parameters: slice thickness, 5 mm; 120 kV, and 100–300 mAs. Images were reviewed in the Digital Imaging and Communications in Medicine (DICOM) format by two board-certified neurologists blinded to the data and not involved in clinical management. Hematoma volume was calculated per protocol on the baseline and stability CT using semi-automatic software (ITK-SNAP software, University of Pennsylvania, Philadelphia, USA; www.itksnap.org). IVH was defined as an intraventricular hyperdense image that was not attributable to the choroid plexus or calcification and was not included in the hematoma volume. Lobar ICH was defined as a hematoma restricted to the frontal, temporal, parietal, or occipital areas. The origin of the hemorrhage appeared at the cortical and subcortical junctions.

### 2.5. Assessment of cSS

All patients underwent an MRI during hospitalization. If data from more than one MRI scan were available, the earliest scan was chosen to retrieve the data (median delay, 5 days; IQR, 4–7 days after ICH). MRI was performed using a 3.0-Telsa scanner (Signa HDxt, GE Healthcare, USA) with a standardized protocol consisting of at least T1, T2, and SWI sequences (axial acquisition plane 3.0 T, TR 5,200 ms, TE 75 ms, b = 0/1,000 s/mm^2^, 6-mm slice thickness, 0.5-mm gap, FOV 240 × 240 mm). In total, two board-certified neurologists assessed the presence of cSS, defined as a curvilinear signal loss on SWI in compliance with the gyral cortical surface within the subarachnoid space, away from at least two sulci of the macrohemorrhage with no corresponding signal hyperintensity on the baseline CT scan (2, 8). We categorized cSS as focal (restricted to ≤3 sulci) or disseminated (affecting at least four sulci) (2).

We also recorded the presence of cerebral microbleeds (CMB), defined as round or ovoid signal voids of 2 and 10 mm in diameter, with associated blooming on the SWI sequences (10–12). CMB locations were categorized as lobar, deep structures (basal ganglia, thalamus, and internal capsule), the brainstem, or the cerebellum. Probable cerebral amyloid angiopathy (CAA) was evaluated according to the modified Boston criteria (13). We evaluated white matter hyperintensity (WMH) visually on axial FLAIR images using the 4-point Fazekas' rating scale. The total WMH score was the addition of the scores for periventricular white matter and deep white matter hyperintensities (11).

### 2.6. Follow-up

The 90-day mRS was obtained through face-to-face or telephone interviews with the patients using standardized questionnaires based on the Rankin Focused Assessment, as described previously (14). At least one telephone number was collected on admission to ensure maximum follow-up integrity, and most patients had two telephone numbers recorded (telephone number of relatives). Patients were defined as lost to follow-up if five or more calls could not contact them. Furthermore, we provided free visits to the Neurology Clinic of the Second Affiliated Hospital of Zhejiang University, School of Medicine, for those unwilling to share recovery conditions. In addition, as the only regional advanced stroke center responsible for quality control, we maintained good contact with other hospitals at different levels in Zhejiang Province. Additionally, we entrusted local doctors with follow-up if necessary.

### 2.7. Statistical analysis

Continuous variables are presented as mean (SD) or median (IQR) and were compared using the *t*-test or the Mann–Whitney *U*-test. Categorical variables were presented as counts (percent) and were analyzed using the Pearson χ^2^ test or Fisher's exact test, as appropriate. Clinical data (demographics, vascular risk factors, medical history, medication and GCS on admission, and imaging characteristics) were compared between the two groups of patients (with vs. without cSS). The same comparisons were made between the patients with focal and disseminated cSS. Hematoma volume and 90-day mRS score were calculated as continuous variables (15). According to published literature and pathophysiological consideration, we first selected clinical information including age, sex, hypertension, diabetes mellitus, atrial fibrillation, coronary heart disease, previous ischemic stroke or TIA, previous ICH, alcohol intake, smoking, antiplatelet drugs, anticoagulant drugs, statin, lobar ICH, intraventricular extension, cSS, CMB, and GCS on admission in univariable analysis. Variables with a *p*-value of <0.1 in univariable analysis were included in multivariable linear regression analyses to determine the risk factors for hematoma volume and 90-day mRS. An additional multivariable regression model was performed to evaluate the relationship between cSS and hematoma volume after discriminating lobar or non-lobar hematoma location.

**Figure 2:**
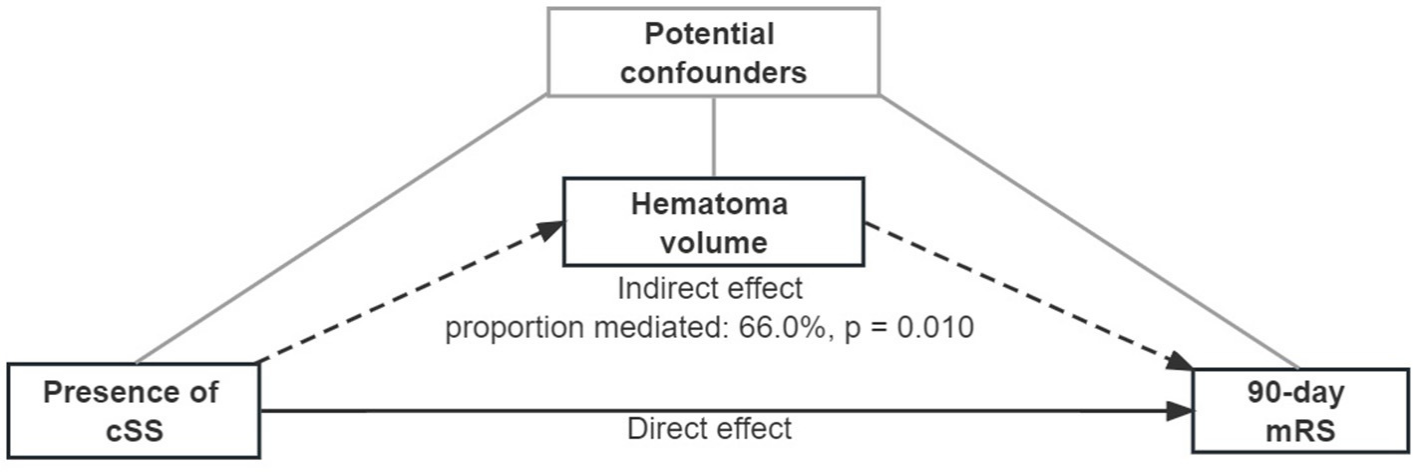
Mediation analysis of associations between cortical superficial siderosis and the 90-day modified Rankin scale, mediated by hematoma volume. A continuous arrow and the indirect effect by dotted arrows represent the direct effect. Adjusted potential confounders (age, sex, previous ICH, intraventricular extension, and GCS score) were selected from the multiple regression model.

Regression-based mediation analyses were then performed to distinguish the direct effect of cSS on 90-day mRS, and the indirect effect mediated by hematoma volume (16). Three estimates were calculated: (i) total effect, which indicated the whole association between cSS and 90-day mRS, composed of direct and indirect effects; (ii) direct effect, disclosing the sole association between cSS itself and 90-day mRS, except for the part caused by the mediator; and (iii) indirect effect, the part of causal relationship mediated by the mediator, that is, hematoma volume, intraventricular hemorrhage extension, and recurrent hemorrhage at present (Figure 2; Supplementary Tables 6, 7). All mediation calculations were non-parametric bootstrapped and estimated at a 95% CI when estimating the coefficients and residuals. Additional analyses were performed for patients with and without probable CAA. A sensitivity analysis was conducted among patients who had a CT scan within 6 h after ICH ictus.

Statistical analyses were performed using Empower^®^ (*http://www.empowerstats.com*; X&Y Solutions, Inc., Boston, MA) and R (https://www.R-project.org/). A *P-*value of <0.05 was considered to be statistically significant. All the significance was two-tailed.

## 3. Results

### 3.1. Characteristics of participants

From the original cohort of 1,044 patients with primary ICH that occurred within 48 h, 673 patients were included in hematoma volume evaluation, and 663 patients were included when evaluating 90-day mRS because 10 patients failed to keep up with the follow-up. The baseline characteristics were compared between the included and excluded patients (Supplementary Table 1).

### 3.2. Prevalence and distribution of cSS

In the entire cohort, 131 out of 673 patients (19.5%) presented with cSS on acute-phase MRI, and disseminated cSS was observed in 29 patients (22.1%). Probable CAA was identified in 48 patients (7.2%). The demographic and clinical characteristics of patients with and without cSS were compared (Table 1). Patients with cSS were older and had a larger hematoma volume, more lobar CMBs, and a higher prevalence of previous ICH, lobar hematoma, intraventricular extension, and probable CAA. In addition, worse neurological presentation and long-term outcomes (GCS on admission and 90-day mRS) were observed in these patients. No significant differences were found in other characteristics, including sex, past medication, and other vascular risk factors (with the exception of previous ICH).

**Table 1:** Comparison of characteristics in patients with and without cortical superficial siderosis.

	**Patients without cSS (*n* = 542)**	**Patients with cSS (*n* = 131)**	***p*-value**
**Demographics**			
Age, mean (SD), year	60 (13)	68 (11)	<0.001
Female sex, *n* (%)	190 (35.1%)	47 (35.9%)	0.860
**Vascular risk factors and medical history**, ***n*** **(%)**			
Hypertension	416 (76.8%)	93 (71.0%)	0.168
Diabetes mellitus	88 (16.2%)	24 (18.3%)	0.565
Atrial fibrillation	14 (2.6%)	7 (5.3%)	0.103
Coronary heart disease	20 (3.7%)	7 (5.3%)	0.387
Ischemic stroke or TIA	49 (9.0%)	13 (9.9%)	0.754
Previous ICH	28 (5.2%)	17 (13.0%)	0.001
Alcohol intake	161 (29.7%)	48 (36.6%)	0.124
Smoking	180 (33.2%)	48 (36.6%)	0.457
**Medication at baseline**, ***n*** **(%)**			
Antiplatelet drugs	46 (8.5%)	12 (9.2%)	0.805
Anticoagulant drugs	7 (1.3%)	0 (0.0%)	/
Statins	27 (5.0%)	9 (6.9%)	0.389
**Imaging characteristics**			
OCT, hour^†^	5.0 (2.0–8.0)	5.0 (3.0–8.5)	0.095
Hematoma volume, ml^†^	8.3 (2.7–16.2)	12.1 (5.2–30.8)	<0.001
Lobar hematoma, *n* (%)	87 (16.1%)	56 (42.7%)	<0.001
Intraventricular extension, *n* (%)	127 (23.4%)	65 (49.6%)	<0.001
OMT, day^†^	5 (4–7)	6 (4–7)	0.011
WMH	2 (1–4)	4 (2–6)	<0.001
CMBs, *n*^†^	2 (0–5)	5 (1–23)	<0.001
Lobar CMBs, *n*^†^	0 (0–2)	2 (0–11)	<0.001
Deep CMBs, *n*^†^	1 (0–3)	1 (0–4)	0.336
**Probable CAA**, ***n*** **(%)**	13 (2.4%)	35 (26.7%)	<0.001
**Functional neurologic evaluation**			
GCS on admission^†^	15 (14–15)	15 (14–15)	0.008
90-day mRS^‡^	1 (0–3)	2 (1–4)	<0.001

TIA, transient ischemic attack; OCT, time from onset to CT; OMT, time from onset to MRI; WMH, white matter hyperintensities; CMB, cerebral microbleed; CAA, cerebral amyloid angiopathy.

^†^Median (interquartile range, Mann–Whitney *U*-test).

^‡^663 patients underwent 90-day mRS evaluation.

The same characteristics were compared between patients with focal and disseminated cSS (Supplementary Table 2). We found that patients with disseminated cSS were older and had a larger hematoma volume, whereas vascular risk factors, medication, and neurological impairment (GCS on admission) did not differ significantly.

### 3.3. cSS and hematoma volume

We performed univariate and multivariate analyses of hematoma volume (Supplementary Table 3; Table 2). Multivariable regression analyses revealed that diabetes mellitus, lobar ICH, intraventricular extension, cSS, and fewer deep CMBs were independently associated with larger hematoma volume. Additionally, in stratified analyses according to hematoma location, cSS was independently associated with a larger hematoma volume in both lobar and non-lobar ICH (Table 2). Hematoma volume remained significantly different compared to patients without cSS after stratification as focal (β = 2.99, 95% CI 0.24–5.73, *p* = 0.033) and disseminated (β = 10.01, 95% CI 5.34–14.68, *p* < 0.001, Supplementary Table 4).

**Table 2:** Multivariable regression analyses of factors associated with hematoma volume stratified by hematoma location.

**Variables**	**Total cohort**		**Non-lobar ICH**		**Lobar ICH**	
	β **(95% CI)**	***p*** **-value**	β **(95% CI)**	***p*** **-value**	β **(95% CI)**	***p*** **-value**
Age	−0.037 (−0.110 to 0.036)	0.322	−0.067 (−0.133 to −0.001)	0.046	0.010 (−0.226 to 0.246)	0.931
Hypertension	0.031 (−1.864 to 2.527)	0.767	0.409 (−1.644 to 2.461)	0.696	3.309 (−2.936 to 9.553)	0.301
Diabetes mellitus	−3.444 (−5.903 to −0.985)	0.006	−2.827 (−4.991 to −0.663)	0.011	−6.707 (−15.415 to 2.000)	0.133
Lobar hematoma	13.787 (11.398–16.176)	<0.001	/		/	
Intraventricular extension	3.702 (1.631–5.773)	<0.001	0.776 (−1.086 to 2.638)	0.414	17.084 (10.406–23.761)	<0.001
cSS	4.449 (1.890–7.009)	<0.001	2.550 (0.033–5.066)	0.048	8.092 (1.689–14.494)	0.014
**CMB**						
Lobar CMBs	0.005 (−0.116 to 0.126)	0.935	−0.018 (−0.197 to 0.160)	0.842	−0.004 (−0.214 to 0.206)	0.971
Deep CMBs	−0.311 (−0.516 to −0.106)	0.003	−0.194 (−0.407 to 0.019)	0.075	−0.615 (−1.306 to 0.077)	0.084

Variables were selected from the univariable analyses with *p* < 0.1 as a screening criterion.

### 3.4. cSS and 90-day mRS

Univariate and multivariate analyses were performed for the 90-day mRS (Supplementary Table 5; Table 3). In total, 663 patients were included in the analysis. Multivariable regression analyses revealed that the characteristics independently associated with worse 90-day mRS were older age, female sex, larger hematoma volume, intraventricular extension, and low GCS score on admission (Table 3). However, cSS was not independently associated with a higher 90-day mRS when adjusted for these covariates, including hematoma volume. After removing hematoma volume from the multivariable analyses, cSS was an independent risk factor for poor outcomes (Table 4).

**Table 3:** Multivariable regression analyses of factors associated with the 90-day modified Rankin scale^†^.

**Variables**	**B**	**95% CI**	***p*-value**
**Multivariable model 1** ^‡^			
Age	0.022	0.013–0.031	<0.001
Female	0.245	−0.008 to 0.482	0.043
Previous ischemic stroke or TIA	0.133	−0.259 to 0.525	0.506
Previous ICH	0.402	−0.055 to 0.859	0.085
Hematoma volume, ml	0.033	0.024–0.042	<0.001
Intraventricular extension	0.262	0.004–0.519	0.047
Lobar CMBs	0.011	−0.002 to 0.025	0.108
cSS	0.074	−0.246 to 0.394	0.650
GCS on admission	−0.173	−0.237 to −0.110	<0.001
**Multivariable model 2** ^§^			
Age	0.022	0.013–0.031	<0.001
Female	0.218	−0.029 to 0.465	0.084
Previous ischemic stroke or TIA	0.110	−0.298 to 0.517	0.598
Previous ICH	0.297	−0.178 to 0.772	0.221
Intraventricular extension	0.303	0.035–0.571	0.027
Lobar CMBs	0.012	−0.002 to 0.026	0.084
cSS	0.333	0.008–0.659	0.045
GCS on admission	−0.220	−0.285 to −0.155	<0.001

^†^663 patients with 90-day follow-up consent for analysis.

^‡^Multivariable model 1: variables were selected from the univariate analyses with a *p*-value <0.1 as a screening criterion.

^§^Multivariable model 2: hematoma volume was removed from the multivariable analyses.

**Table 4:** Mediation analyses of the association between cortical superficial siderosis and the 90-day modified Rankin scale mediated by hematoma volume.

**Mediation analyses**	**Parameter estimate**	**95% CI**	***p*-value**
**Model 1: in the whole cohort (*****n*** = **663)**^†^			
Direct effect	0.138	−0.212 to 0.510	0.468
Mediation effect	0.269	0.145–0.420	<0.001
Total effect	0.408	0.074–0.754	0.010
Proportion mediated (%)	66.04	28.67–311.19	0.010
**Model 2: in patients with probable CAA (*****n*** = **47)**^‡^			
Direct effect	1.553	0.410–2.808	0.008
Mediation effect	0.264	−0.196 to 0.781	0.244
Total effect	1.817	0.844–2.852	0.004
Proportion mediated (%)	14.51	−9.28 to 55.96	0.248
**Model 3: in patients without probable CAA (*****n*** =**616)**^§^			
Direct effect	0.189	−0.171 to 0.541	0.360
Mediation effect	0.182	0.045–0.361	0.002
Total effect	0.371	0.019–0.732	0.040
Proportion mediated (%)	48.97	4.65–347.96	0.042

Adjusted for age, sex, previous ICH, GCS, and intraventricular extension.

^†^663 patients who had a 90-day mRS evaluation consented to mediation analyses.

^‡^47 out of 48 patients with probable CAA underwent a 90-day mRS evaluation.

^§^616 of 625 patients without probable CAA underwent a 90-day mRS evaluation.

The distribution of 90-day mRS scores is presented in Supplementary Figure 1, in which data from patients with and without cSS, and with focal or disseminated cSS were compared.

### 3.5. Mediation analysis

Based on the analysis of cSS with hematoma volume and 90-day mRS, we tested the hypothesis that hematoma volume might be an essential mediator between cSS occurrence and unfavorable 90-day outcomes (Figure 2; Table 4). The association between cSS and unfavorable outcomes can be subdivided into a direct effect of cSS and an indirect effect mediated by large hematoma volume induced by cSS. It revealed that the association between cSS and worse 90-day outcomes was mainly mediated by induction of a larger hematoma volume (proportion mediated: 66.0%, *p* = 0.010), whereas the direct effect of cSS on 90-day mRS was not significant.

Additional mediation analyses were performed for other probable factors, including probable CAA, previous ICH, and intraventricular extension. In patients without probable CAA, the mediation effect was consistent with that in the whole cohort (proportion mediated: 49%, *p* = 0.042, Table 4). Recurrent ICH showed an unremarkable mediation effect, whereas intraventricular extension presented a minor effect (proportion mediated: 15.8%, *p* = 0.044) on cSS and 90-day mRS (Supplementary Tables 6, 7).

The sensitivity analysis including 449 patients who had a CT scan within 6 h after ICH ictus suggested that hematoma volume was still a significant mediator between cSS and 90-day outcome (proportion mediated: 68.1%, *p* = 0.006); the direct effect of cSS on 90-day mRS was not remarkable (Supplementary Table 8).

For WMH, we performed univariable analysis for hematoma volume and found that WMH was not associated with large hematoma volume (β = −0.342, 95% CI −0.920–0.235, *p* = 0.246; Supplementary Table 3), and therefore, WMH should not be included in multivariable regression analysis. With regard to 90-day mRS, WMH had a *p*-value of 0.001 in univariable analysis (β = 0.124, 95% CI 0.053–0.196, *p* = 0.001; Supplementary Table 5) but was not associated with worse 90-day mRS in multivariable regression analysis (β = −0.044, 95% CI −0.125 to −0.037, *p* = 0.287; Supplementary Table 9). We also performed mediation analysis after including WMH as an adjusted factor, and the results showed that hematoma volume remained a significant mediator (proportion mediated: 55.09%, *p* = 0.010; Supplementary Table 10).

## 4. Discussion

In this prospective cohort of 673 patients with spontaneous ICH, cSS was observed in one out of five patients. The presence of cSS led to a large hematoma volume, independent of hematoma location and unfavorable 90-day outcomes. The relationship between cSS and 90-day mRS was mainly mediated by a large hematoma volume, as identified in this study, while the sole effect of cSS on 90-day mRS was unremarkable.

For decades, cSS has been proven to be a strong risk factor for unfavorable neurologic outcomes and is related to other clinical characteristics that correlate with worse outcomes, such as recurrent ICH and large hematoma (1, 3–8). However, it is unclear whether the deleterious outcome is determined by cSS or by other potential factors induced by cSS. We made the hypothesis that part of the effect (indirect effect) of cSS and 90-day mRS was mediated by hematoma volume based on the results of numerous cohort studies that concluded those as follows: (i) cSS is associated with large hematoma volume in patients with ICH; (ii) patients with cSS are more likely to have poor outcomes after ICH, including higher risk of recurrent ICH and poor functional recovery; and (iii) hematoma volume is the most significant factor deciding prognosis of ICH, and large hematoma volume leads to poor outcomes (1, 3–8). Therefore, a probable relationship chain is that cSS leads to large hematoma volume and then caused poor outcomes. We used mediation analysis to test this hypothesis and identified large hematoma as the essential pathway leading to worse neurologic recovery because of cSS and quantified the prominent mediation effect.

In our study, cSS was related to large hematoma in both lobar and non-lobar areas. This contradicts previous findings reported on patients from Western countries, indicating that cSS is mainly associated with large lobar hemorrhage (1). In *in vivo* and *ex vivo* MRI examinations and neuropathological studies on CAA-related patients, most affected arteries tended to be located in the intra-sulcal part of the hematoma. Thus, the origin of cSS appeared from the meningeal arteriole, with larger diameters than those in bulging areas on the cortex (17, 18). It was speculated that successive vessel ruptures in the sulci initiated the formation of an intra-sulcal hematoma, accompanied by surrounding hemorrhagic or anemic infarct in the cerebral cortex, which then extended into the brain parenchyma and generated a lobar ICH. The presence of cSS, therefore, emerges as a history of leakage of blood from small vessels located in the sulci, also indicating a higher risk of larger hematoma volume and hematoma expansion into brain parenchyma (17). The affected vessel tended to have a meningeal rather than cortical location, which was assumed to be because of APOE e2genotype of, assumed to aggravate vessel fragility and enhanced blood leakage (19). However, in this study, including Asian patients with primary ICH, we found that cSS was not only related to a larger hematoma in the lobar area but also in non-lobar areas, which may suggest an underlying etiology other than CAA of cSS in Asian patients (Supplementary Figure 2). This result might be explained by recent evidence testing imaging-diagnosed CAA-probable patients with PiB-PET in Taiwan, which found that in Asian populations, imaging markers indicating CAA may display a much lower predictive effect (20).

Although both are recognized as indicators of chronic blood extravasation in the brain, cSS showed clinical characteristics as opposed to CMB. Recent cohort studies have reported an inverse relationship between CMB and ICH volume and hematoma expansion, regardless of CMB location (lobar or deep) (1, 10). The results of CMB in our cohort presented similar results. We found that patients with higher CMB counts had smaller ICH volumes at admission. However, the results of our study were different from those previously reported in patients with cSS (1). This interesting difference between CMB and cSS suggests different mechanisms regulating ICH formation and hematoma enlargement, which warrants further exploration.

In the entire cohort, 48 patients (7.2%) were diagnosed with probable CAA according to the modified Boston criteria. Surprisingly, the mediation analyses in patients with and without probable CAA identified hematoma volume as an important mediator for 90-day mRS only in patients without probable CAA. We speculate that patients with probable CAA had more extensive and profound destruction of vessels and brain structure caused by CAA, which led to a series of pathological statuses and might fundamentally affect long-term recovery; thus, neurological dysfunction may be integrated with other noxious effectors, such as dementia and depression, in this particular case. Earlier studies also reported that dementia and depression were common in CAA patients, which is independent of ICH occurrence and recurrence (21–27). Herein, we inferred that these deleterious aspects of CAA might take over the central role of hematoma volume, leading to unfavorable outcomes.

Nevertheless, this study has some limitations. In this single-center cohort study in Hangzhou, China, the proportion of patients with lobar ICH was lower than that reported in other studies in the European cohort (8). Thus, the proportion of patients with probable CAA was low. The etiologies of these patients are quite different from those found in patients with ICH from Western countries. Second, cSS was assessed on MRI with a median delay of 5 (IQR, 4–7) days after ICH, but not before ICH ictus. The assumption is that cSS was chronic or former minimum leakage of blood before ICH, and we identified gyral high density on CT to exclude the newly formed cortical hemorrhage (2, 28). In addition, mRS was analyzed as a continuous variable in this study to retain the complete information in mediation analyses; this is less common but has been proven feasible in a previous article (15). We excluded patients with surgical procedures, mRS ≥ 3 before ICH, and those without MRI during hospitalization. Patients did not undergo MRI if they had a pacemaker, prosthetic heart valve, severe neurologic impairment, and unstable vital signs. Therefore, the severity of the patients included in this study was relatively mild; this explains why the patients had better outcomes than those in other studies (29–31).

## 5. Conclusion

This study highlighted the critical role of hematoma volume as a mediator between cSS and poor outcomes after mild to moderate ICH. In contrast, the noxious effect of a large hematoma because of cSS was independent of hematoma location, either in lobar or non-lobar areas.

## Data availability statement

The raw data supporting the conclusions of this article will be made available by the authors, without undue reservation.

## Ethics statement

The studies involving human participants were reviewed and approved by the Human Investigation Committee (IRB) of Second Affiliated Hospital of Zhejiang University. Written informed consent for participation was not required for this study in accordance with the national legislation and the institutional requirements.

## Author contributions

Y-jJ and L-sT: conceptualization. Y-jJ, Y-hH, and C-zY: methodology. J-wL, JW, K-cY, and H-nA: formal analysis and investigation. Y-jJ: writing—original draft preparation. L-sT and FG: writing—review and editing. L-sT: funding acquisition. FG: resources. J-wL, L-sT, and FG: supervision. All authors contributed to the article and approved the submitted version.
