# Supplementary material for: Cortical superficial siderosis, hematoma volume, and outcomes after intracerebral hemorrhage: a mediation analysis
Source: Front Neurol. 2023 May 5;14:1122744. doi: 10.3389/fneur.2023.1122744 (PMC10196120; doi:10.3389/fneur.2023.1122744)
Supplement: Supplementary file 1 [file Data_Sheet_1.docx]

Supplemental materials

| **Contents** | **Page numbers** |  |
| --- | --- | --- |
| **eTable 1** Comparison of characteristics in patients included and excluded | 1 |  |
| **eTable 2** Comparison of characteristics in patients with focal and disseminated cortical superficial siderosis | 2 |  |
| **eTable 3** Univariable regression analyses of factors associated with hematoma volume | 3 |  |
| **eTable 4** Multivariable regression analyses of factors associated with hematoma volume after classifying cSS as focal and disseminated. | 4 |  |
| **eTable 5** Univariable regression analyses of factors associated with 90-day modified Rankin scale | 5 |  |
| **eFigure 1** 90-day modified Rankin scale distribution | 6 |  |
| **eTable 6** Mediation analyses of the association between cortical superficial siderosis and 90-day modified Rankin scale mediated by recurrent intracerebral hemorrhage | 7 |  |
| **eTable 7** Mediation analyses of the association between cortical superficial siderosis and 90-day modified Rankin scale mediated by intraventricular extension | 8 |  |
| **eTable 8** Mediation analyses of the association between cortical superficial siderosis and 90-day modified Rankin scale mediated by hematoma volume among patients who had CT scan within 6 hours after ICH ictus | 9 | |
| **eFigure 2** cSS and deep-located ICH. | 10 |  |
| **eTable 9** Multivariable regression analyses of factors associated with 90-day modified Rankin scale (WMH included)†. | 11 |  |
| **eTable 10** Mediation analyses of the association between cortical superficial siderosis and 90-day modified Rankin scale mediated by hematoma volume with WMH as an adjusted factor. | 12 | |

**eTable 1** Comparison of characteristics in patients included and excluded

|  | **Patients included**  **(n=673)** | **Patients excluded**  **(n=371)** | ***p* Value** |  |  |
| --- | --- | --- | --- | --- | --- |
| **Demographics** | |  |  | |  |
| Age, mean (SD), year | 61 (13) | 62 (16) | 0.108 | |  |
| Female sex, n (%) | 237 (35.2%) | 109 (33.1%) | 0.515 | |  |
| **Vascular risk factors and medical history, n(%)** | | | | | |
| Hypertension | 509 (75.6%) | 175 (74.2%) | 0.651 | | |
| Diabetes mellitus | 112 (16.6%) | 34 (14.5%) | 0.435 | | |
| Atrial fibrillation | 21 (3.1%) | 19 (8.1%) | 0.001 | | |
| Coronary heart disease | 27 (4.0%) | 16 (6.9%) | 0.080 | | |
| Ischemic stroke or TIA | 27 (4.0%) | 22 (9.4%) | 0.931 | | |
| Recurrent ICH | 45 (6.7%) | 17 (7.3%) | 0.751 | | |
| Alcohol intake | 209 (31.1%) | 88 (37.3%) | 0.079 | | |
| Smoking | 228 (33.9%) | 89 (37.8%) | 0.288 | | |
| **Medication at baseline, n(%)** | | |  | | |
| Antiplatelet drugs | 58 (8.6%) | 29 (12.3%) | 0.099 | | |
| Anticoagulant drugs | 7 (1.0%) | 16 (6.8%) | <0.001 | | |
| Statin | 36 (5.3%) | 21 (8.9%) | 0.051 | | |
| **GCS on admission**† | 15 (14-15) | 14 (11-15) | <0.001 | | |
| **Imaging characteristics** | | | | | |
| OCT, hour† | 5 (2-8) | 7 (3-24) | <0.001 | | |
| Hematoma volume, ml† | 9.0 (3.0-17.6) | 10.5 (4.2-26.4) | 0.007 | | |
| Lobar ICH, n (%) | 143 (21.2%) | 56 (25.6%) | 0.033 | | |
| Intraventricular extension, n (%) | 192 (28.5%) | 86 (39.8%) | 0.002 | | |
| OMT, day† | 5 (4-7) | 5 (3-7) | 0.102 | | |

Abbreviations: TIA = transient ischemic attack; ICH = intracerebral hemorrhage; GCS = [Glasgow Coma Scale](https://www.mdcalc.com/glasgow-coma-scale-score-gcs); OCT = time from onset to first CT; OMT = time from onset to MRI.

†Median (interquartile range, Mann-Whitney test).

**eTable 2** Comparison of characteristics in patients with focal and disseminated cortical superficial siderosis

|  | **Focal cSS**  **(n=102)** | **Disseminated cSS**  **(n=29)** | ***p* Value** |  |  |
| --- | --- | --- | --- | --- | --- |
| **Demographics** | |  |  | |  |
| Age, mean (SD), year | 67 (11) | 72 (12) | 0.015 | |  |
| Female sex, n (%) | 37 (36.3%) | 10 (34.5%) | 0.859 | |  |
| **Vascular risk factors and medical history, n(%)** | | | | | |
| Hypertension | 74 (72.5%) | 19 (65.5%) | 0.462 | | |
| Diabetes mellitus | 21 (20.6%) | 3 (10.3%) | 0.208 | | |
| Atrial fibrillation | 6 (5.9%) | 1 (3.4%) | 1.000 | | |
| Coronary heart disease | 6 (5.9%) | 1 (3.4%) | 1.000 | | |
| Ischemic stroke or TIA | 12 (11.8%) | 1 (3.4%) | 0.186 | | |
| Recurrent ICH | 13 (12.7%) | 4 (13.8%) | 0.882 | | |
| Alcohol intake | 38 (37.3%) | 10 (34.5%) | 0.785 | | |
| Smoking | 36 (35.3%) | 12 (41.4%) | 0.548 | | |
| **Medication at baseline, n(%)** | | |  | | |
| Antiplatelet drugs | 9 (8.8%) | 3 (10.3%) | 0.802 | | |
| Anticoagulant drugs | 0 (0%) | 0 (0%) | / | | |
| Statin | 7 (6.9%) | 2 (6.9%) | 1.000 | | |
| **Imaging characteristics** | | | | | |
| OCT, hour† | 5 (3-8) | 6 (3-10) | 0.536 | | |
| Hematoma volume, ml† | 11.0 (4.0-25.8) | 17.4 (9.6-40.2) | 0.014 | | |
| Lobar ICH, n (%) | 43 (42.2%) | 13 (44.8%) | 0.798 | | |
| Intraventricular extension, n (%) | 47 (46.1%) | 18 (62.1%) | 0.129 | | |
| OMT, day† | 6 (4-7) | 7 (5-10) | 0.002 | | |
| Total CMBs, n† | 6 (1-24) | 4 (1-21) | 0.622 | | |
| **Neurologic impairment** |  |  |  | | |
| GCS on admission† | 15 (14-15) | 15 (13-15) | 0.611 | | |
| 90-day mRS† | 2 (0-4) | 3 (1-4) | 0.160 | | |

Abbreviations: cSS = cortical superficial siderosis; TIA = transient ischemic attack; ICH = intracerebral hemorrhage; OCT = time from onset to CT; OMT = time from onset to MRI; CMB = cerebral microbleed; GCS = [Glasgow Coma Scale](https://www.mdcalc.com/glasgow-coma-scale-score-gcs); mRS = modified Rankin scale.

†Median (interquartile range, Mann-Whitney test).

**eTable 3** Univariable regression analyses of factors associated with hematoma volume

| **Variables** | | **β** | | **95% CI** | | ***p* Value** | |
| --- | --- | --- | --- | --- | --- | --- | --- |
| Age | | 0.071 | | -0.009-0.152 | | 0.081 | |
| Female | | 0.498 | | -2.661-1.664 | | 0.652 | |
| Hypertension | | -3.221 | | -5.615- -0.827 | | 0.008 | |
| Diabetes mellitus | | -3.829 | | -6.587- -1.071 | | 0.007 | |
| Atrial fibrillation | | -1.257 | | -7.197-4.684 | | 0.678 | |
| Coronary heart disease | | -3.661 | | -8.918-1.596 | | 0.173 | |
| Ischemic stroke or TIA | | 0.311 | | -3.261-3.883 | | 0.864 | |
| Recurrent ICH | | -1.274 | | -5.408-2.861 | | 0.546 | |
| Alcohol intake | | 0.242 | | -1.991-2.474 | | 0.832 | |
| Smoking | | 0.710 | | -1.472-2.892 | | 0.524 | |
| Antiplatelet drugs | | -1.103 | | -4.783-2.577 | | 0.557 | |
| Anticoagulant drugs | | -1.695 | | -11.877-8.486 | | 0.744 | |
| Statin | | -0.289 | | -6.678-2.499 | | 0.372 | |
| Lobar ICH | | 14.716 | | 12.450-16.983 | | <0.001 | |
| Intraventricular extension | | 3.772 | | 1.502-6.042 | | 0.001 | |
| cSS | | 8.316 | | 5.784-10.848 | | <0.001 | |
| Focal cSS | | 5.638 | | 2.789-8.487 | | 0.001 | |
| Disseminated cSS | | 14.033 | | 9.058-19.009 | | <0.001 | |
| Total CMBs | | 0.016 | | -0.059-0.092 | | 0.670 | |
| Lobar CMBs | | 0.140 | | 0.023-0.256 | | 0.019 | |
| Deep CMBs | | -0.284 | | -0.490- -0.077 | | 0.007 | |
| WMH | | -0.342 | | -0.920-0.235 | | 0.246 | |

Abbreviations: mRS = modified Rankin scale; TIA = transient ischemic attack; ICH = intracerebral hemorrhage; cSS = cortical superficial siderosis; CMB = cerebral microbleed.

**eTable 4** Multivariable regression analyses of factors associated with hematoma volume.

| **Variables** | **β** | **95% CI** | ***p* Value** |
| --- | --- | --- | --- |
| Age | -0.046 | -0.119- 0.028 | 0.222 |
| Hypertension | 0.358 | -1.826-2.543 | 0.748 |
| Diabetes mellitus | -3.251 | -5.701- -0.800 | 0.010 |
| Lobar hematoma | 13.779 | 11.402-16.156 | <0.001 |
| Intraventricular extension | 3.512 | 1.447-5.577 | <0.001 |
| cSS |  |  |  |
| Focal cSS | 2.988 | 0.241-5.734 | 0.033 |
| Disseminated cSS | 10.007 | 5.335-14.679 | <0.001 |
| CMBs |  |  |  |
| Lobar CMBs | 0.005 | -0.115-0.125 | 0.937 |
| Deep CMBs | -0.290 | -0.494- -0.085 | 0.006 |

Variables were selected from the univariable analyses with p < 0.1 as a screening criterion.

**eTable 5** Univariable regression analyses of factors associated with 90-day modified Rankin scale†.

| **Variables** | | **β** | | **95% CI** | | ***p* Value** | |
| --- | --- | --- | --- | --- | --- | --- | --- |
| Age | | 0.030 | | 0.021-0.040 | | <0.001 | |
| Female | | 0.298 | | 0.035-0.561 | | 0.027 | |
| Hypertension | | -0.131 | | -0.424-0.162 | | 0.381 | |
| Diabetes mellitus | | -0.186 | | -0.522-0.149 | | 0.277 | |
| Atrial fibrillation | | -0.301 | | -1.020-0.417 | | 0.411 | |
| Coronary heart disease | | -0.199 | | -0.836-0.437 | | 0.540 | |
| Ischemic stroke or TIA | | 0.397 | | -0.035-0.828 | | 0.072 | |
| Recurrent ICH | | 0.518 | | 0.019-1.017 | | 0.042 | |
| Alcohol intake | | -0.058 | | -0.330-0.214 | | 0.676 | |
| Smoking | | -0.218 | | -0.483-0.048 | | 0.108 | |
| Antiplatelet drugs | | 0.340 | | -0.105-0.785 | | 0.135 | |
| Anticoagulant drugs | | 0.186 | | -1.045-1.418 | | 0.767 | |
| Statin | | 0.111 | | -0.444-0.667 | | 0.695 | |
| Hematoma volume | | 0.041 | | 0.032-0.050 | | <0.001 | |
| Lobar ICH | | 0.161 | | -0.147-0.470 | | 0.305 | |
| Intraventricular extension | | 0.585 | | 0.310-0.859 | | <0.001 | |
| cSS | |  | |  | |  | |
| Focal cSS | | 0.585 | | 0.237-0.932 | | 0.001 | |
| Disseminated cSS | | 1.088 | | 0.467-1.708 | | <0.001 | |
| CMBs | |  | |  | |  | |
| Lobar CMBs | | 0.027 | | 0.013-0.041 | | <0.001 | |
| Deep CMBs | | 0.006 | | -0.019-0.032 | | 0.617 | |
| WMH | | 0.124 | | 0.053-0.196 | | 0.001 | |
| GCS on admission | | -0.251 | | -0.318- -0.184 | | <0.001 | |

Abbreviations: TIA = transient ischemic attack; ICH = intracerebral hemorrhage; cSS = cortical superficial siderosis; CMB = cerebral microbleed; GCS = [Glasgow Coma Scale](https://www.mdcalc.com/glasgow-coma-scale-score-gcs).

†663 patients with 90-day follow-up consented for analysis.

**eFigure 1** 90-day modified Rankin scale distribution.


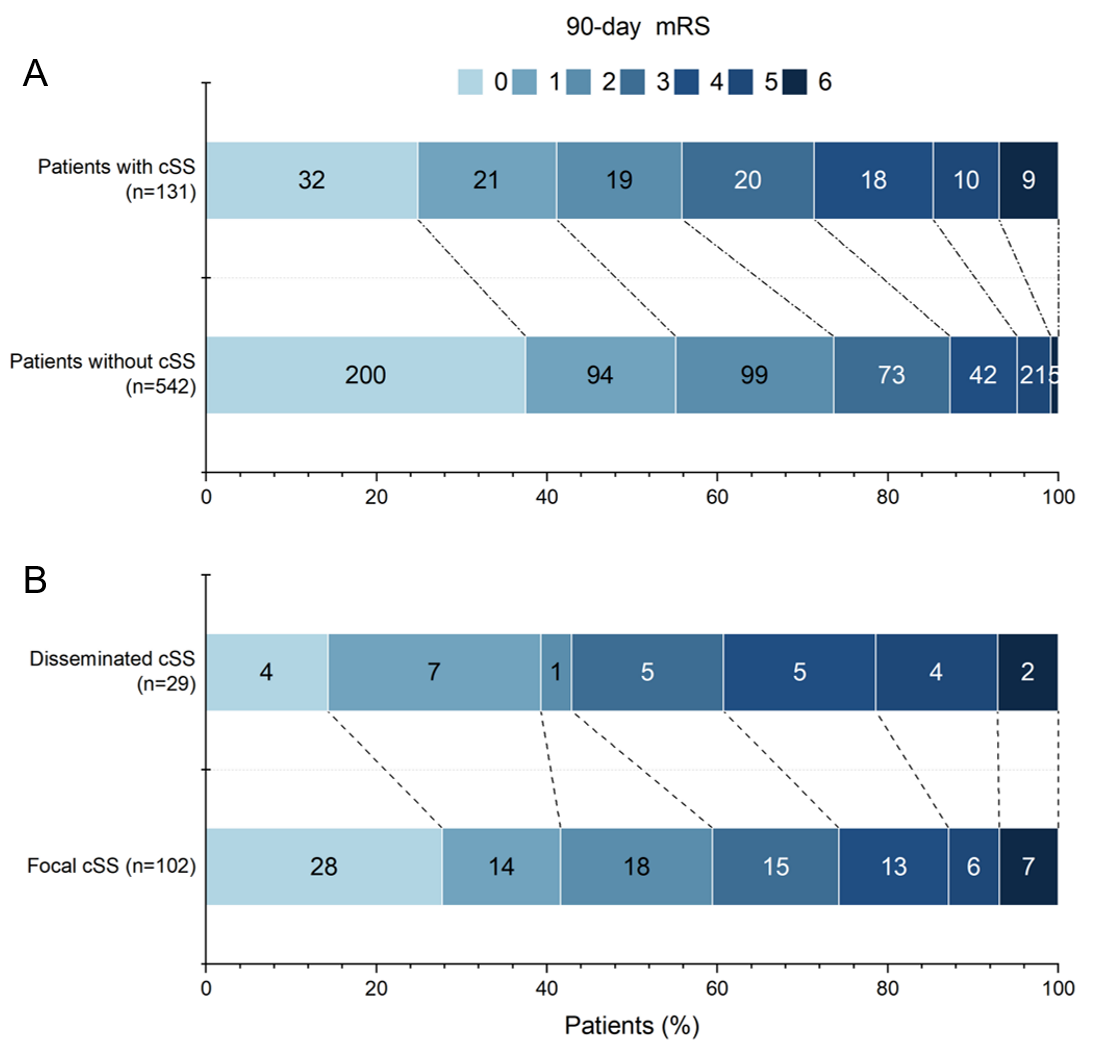


eFig1-A showed distribution of 90-day mRS in patients with and without cSS. eFig1-B showed distribution of 90-day mRS in patients with focal and disseminated cSS.

**eTable 6** Mediation analyses of the association between cortical superficial siderosis and 90-day modified Rankin scale mediated by recurrent intracerebral hemorrhage

| **Mediation analyses**† | **Parameter estimate** | | **95% CI** | **P value** |
| --- | --- | --- | --- | --- |
| Direct effect | 0.478 | 0.074-0.754 | | 0.010 |
| Mediation effect | 0.021 | -0.012-0.097 | | 0.190 |
| Total effect | 0.429 | 0.112-0.782 | | 0.002 |
| Proportion mediated (%) | 4.93 | -3.47-41.46 | | 0.192 |

Adjusted for age, sex, GCS and intraventricular extension.

†663 patients who had 90-day mRS evaluation consented for mediation analyses.

**eTable 7** Mediation analyses of the association between cortical superficial siderosis and 90-day modified Rankin scale mediated by intraventricular extension

| **Mediation analyses**† | **Parameter estimate** | | **95% CI** | **P value** |
| --- | --- | --- | --- | --- |
| Direct effect | 0.408 | 0.074-0.754 | | 0.010 |
| Mediation effect | 0.077 | 0.004-0.156 | | 0.044 |
| Total effect | 0.485 | 0.139-0.824 | | <0.001 |
| Proportion mediated (%) | 15.82 | 1.05-55.15 | | 0.044 |

Adjusted for age, sex, GCS and recurrent ICH.

†663 patients who had 90-day mRS evaluation consented for mediation analyses.

**eTable 8** Mediation analyses of the association between cortical superficial siderosis and 90-day modified Rankin scale mediated by hematoma volume among patients who had CT scan within 6 hours after ICH ictus

| **Mediation analyses**† | **Parameter estimate** | | **95% CI** | **P value** |
| --- | --- | --- | --- | --- |
| Direct effect | 0.171 | -0.255-0.608 | | 0.454 |
| Mediation effect | 0.364 | 0.190-0.580 | | <0.001 |
| Total effect | 0.535 | 0.129-0.985 | | 0.006 |
| Proportion mediated (%) | 68.08 | 29.31-259.94 | | 0.006 |

Adjusted for age, sex, previous ICH, GCS and intraventricular extension.†449 patients had CT scan within 6 hours after ICH ictus.

**eFigure 2** cSS and deep-located ICH.


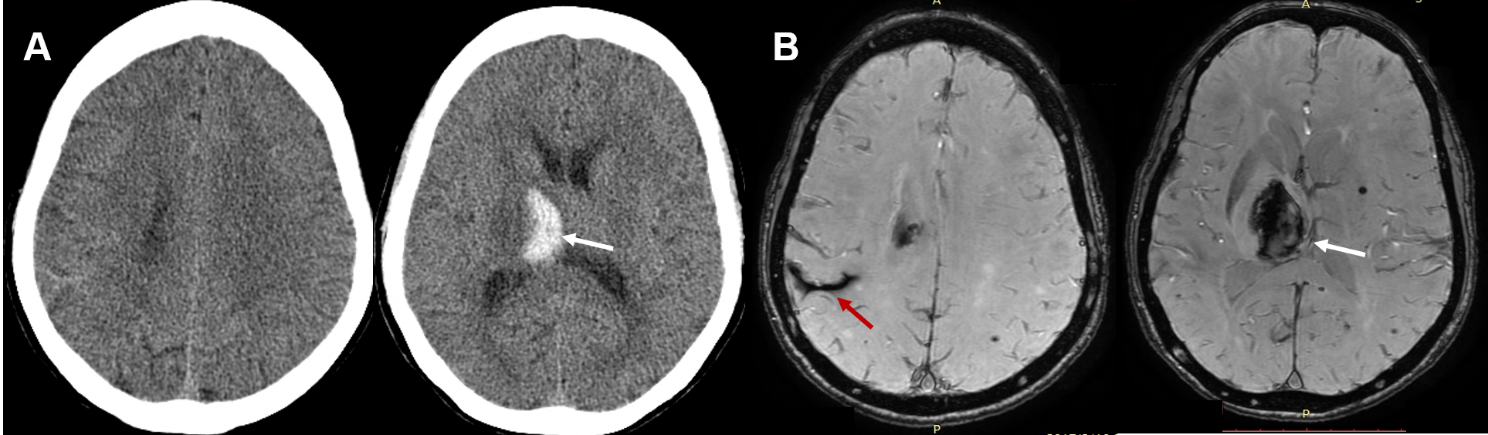


(A) Non-contrast CT scan on admission, showing hemorrhage in right thalamus (white arrow) in a 68-year-old woman. (B) Brain MRI, susceptibility weighted image (SWI) sequences four days later, showing hemorrhage in right thalamus (white arrow) and cortical superficial siderosis (cSS; red arrow).

**eTable 9** Multivariable regression analyses of factors associated with 90-day modified Rankin scale (WMH included)†.

| **Variables** | **Β** | **95% CI** | ***p* Value** |
| --- | --- | --- | --- |
| Age | 0.024 | 0.013-0.035 | <0.001 |
| Female | 0.194 | -0.077-0.465 | 0.161 |
| Previous ischemic stroke or TIA | 0.056 | -0.419-0.531 | 0.818 |
| Previous ICH | 0.041 | -0.470-0.552 | 0.876 |
| Intraventricular extension | 0.319 | 0.032-0.606 | 0.030 |
| Lobar CMBs | 0.015 | -0.000-0.030 | 0.048 |
| cSS | 0.417 | 0.053-0.780 | 0.025 |
| WMH | -0.044 | -0.125-0.037 | 0.287 |
| GCS on admission | -0.215 | -0.285- -0.144 | <0.001 |

†552 patients who had WMH evaluation consented to multivariable regression analysis.

**eTable 10** Mediation analyses of the association between cortical superficial siderosis and 90-day modified Rankin scale mediated by hematoma volume with WMH as an adjusted factor. †

| **Mediation analyses**† | **Parameter estimate** | | **95% CI** | **P value** |
| --- | --- | --- | --- | --- |
| Direct effect | 0.223 | -0.156-0.643 | | 0.228 |
| Mediation effect | 0.283 | 0.132-0.467 | | <0.001 |
| Total effect | 0.506 | 0.160-0.899 | | 0.010 |
| Proportion mediated (%) | 55.90 | 29.29-167.96 | | 0.010 |

Adjusted for age, sex, previous ICH, WMH, GCS and intraventricular extension.

†552 patients had CT scan within 6 hours after ICH ictus.
